# Tracheal Deviation and Airway Management: Clinical Considerations From a Cadaver

**DOI:** 10.7759/cureus.55546

**Published:** 2024-03-05

**Authors:** Takutoshi Inoue, Toru Yamamoto

**Affiliations:** 1 Department of Anatomy, Teikyo University School of Medicine, Tokyo, JPN; 2 Division of Dental Anesthesiology, Graduate School of Medicine and Dental Sciences, Niigata University, Niigata, JPN

**Keywords:** cadaver, carotid tortuosity, airway management, tracheal deviation, anatomy training body

## Abstract

Severe tracheal deviation detected on preoperative chest radiographs is one of the risk factors for difficult tracheal intubation and difficulty in ventilation using an endotracheal tube after tracheal intubation when managing the airway through tracheal intubation under general anesthesia. In this report, we describe the cadaver of an 81-year-old woman with marked tracheal deviation due to meandering multiple aortas. This report details the importance of anatomical knowledge in developing a detailed airway management plan. The deviated trachea was removed from the cadaver and the tracheal tube was inserted at the glottis to the proximal end of the glottal marker. The tube tip was in contact with the tracheal wall, suggesting ventilation difficulty during intubation. The tortuous brachiocephalic artery passed in front of the trachea, which posed a risk of massive aortic hemorrhage and postoperative trachea-brachiocephalic artery fistula during percutaneous tracheostomy for emergency airway management. The anatomical location of the trachea and carotid artery must be confirmed before surgery/anesthesia to ensure safe airway management.

## Introduction

Tracheal deviation is sometimes seen on preoperative chest radiographs. Tracheal deviation is defined as trachea shifts to one side from its normal position in the neck or chest. There are several causes of tracheal deviation, including thyroid disease [1] and meandering of the aortic arch (AA) [2]. Although the epidemiological frequency of occurrence is unclear, severe tracheal deviation during tracheal intubation under general anesthesia is a risk factor for difficult intubation during induction of general anesthesia [3,4] and difficulty in ventilation with an endotracheal tube after tracheal intubation [5,6]. Here, we report a case of tracheal deviation due to the meandering of multiple aortas during anatomical practice at Teikyo University School of Medicine in 2023 along with some findings.

## Case presentation

This case presentation was performed in accordance with the requirements of the Declaration of Helsinki. The authors followed the guidelines for the research involving cadavers established by the Japanese Association of Anatomists for this report.

**Figure 1:**
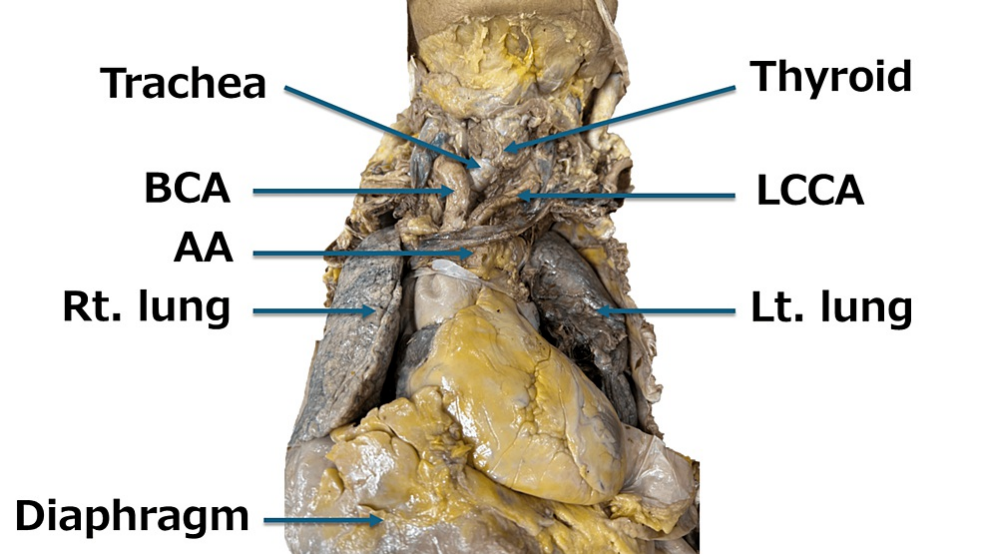
The heart and its surroundings (front). When the thoracic cavity was opened, hypertrophy of the left ventricle (LVH) with a cardiothoracic ratio (CTR) exceeding 50% was first observed. Next, a meandering brachiocephalic artery (BCA) passed across the anterior wall of the trachea, narrowing between the thyroid and BCA. In addition to this, the aortic arch (AA), left common carotid artery (LCCA), left lung (Lt. lung), and right lung (Rt lung) were also observed.

**Figure 2:**
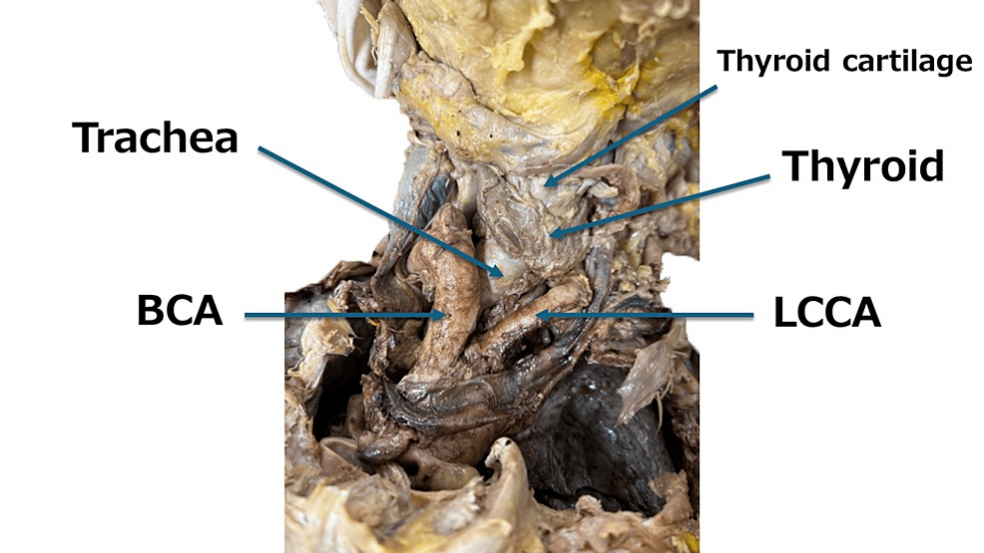
Head and neck arteries and trachea (front). A magnified image of the head and neck (front) is shown. BCA: brachiocephalic artery; LCCA: left common carotid artery

**Figure 3:**
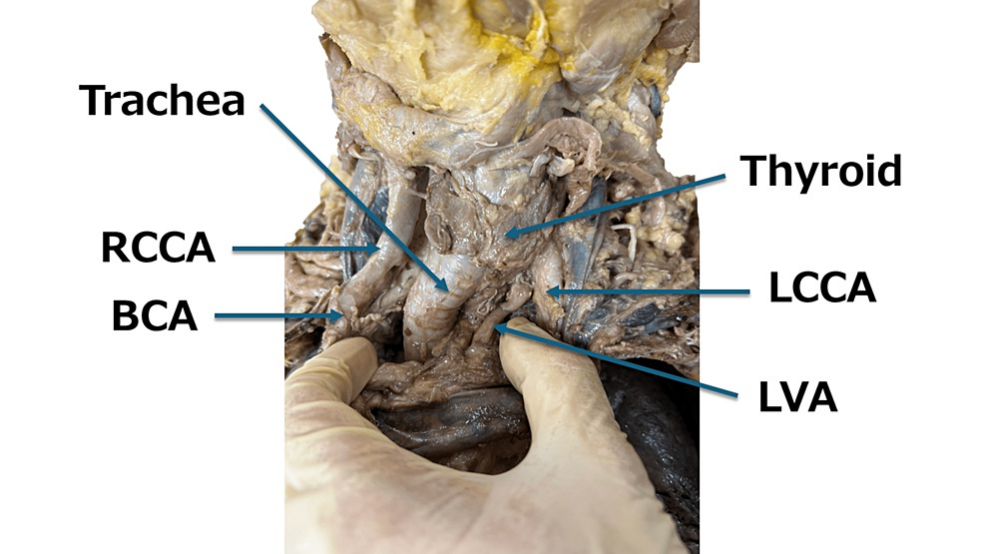
Head and neck arteries and trachea (front). To confirm tracheal deviation, the brachiocephalic artery (BCA) and left common carotid artery (LCCA) were retracted in the lateral direction. As a result, not only tracheal deviation but also meandering of the right common carotid artery (RCCA) was observed. LVA: left vertebral artery

This case report involved an 81-year-old female with a height of 155 cm, weight of 70 kg, and body mass index of 29.1 kg/m^2^. The main cause of death was hepatocellular carcinoma, and the subject had indications of cardiac hypertrophy. No other information regarding prenatal history was available. However, hepatocellular carcinoma suggested hepatitis or liver cirrhosis. For anatomy practice, formalin was injected through the left femoral artery and then the subject was embalmed with formalin and alcohol. While opening and dissecting the thoracic cavity (Figure 1), we observed that the trachea was just above the AA and had deviated significantly to the right. The brachiocephalic artery (BCA) and right common carotid artery (RCCA) were tortuous and the BCA meandered across the anterior wall of the trachea (Figures 2, 3).

**Figure 4:**
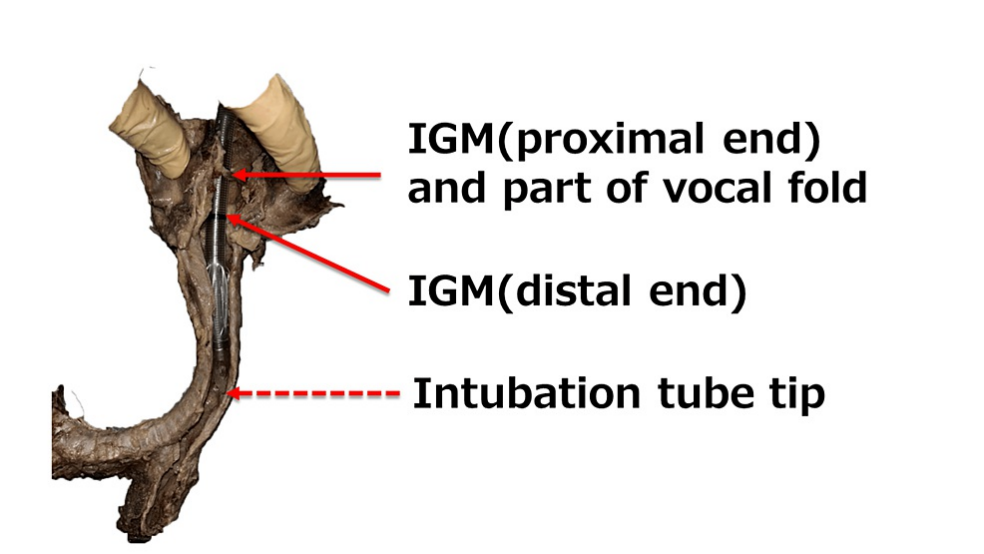
Trachea and intubation tube 1. When the tube was fixed at the proximal end of the glottis marker (IGM), the tip of the intubation tube contacted the tracheal wall. IGM: Intubation Guide Mark

**Figure 5:**
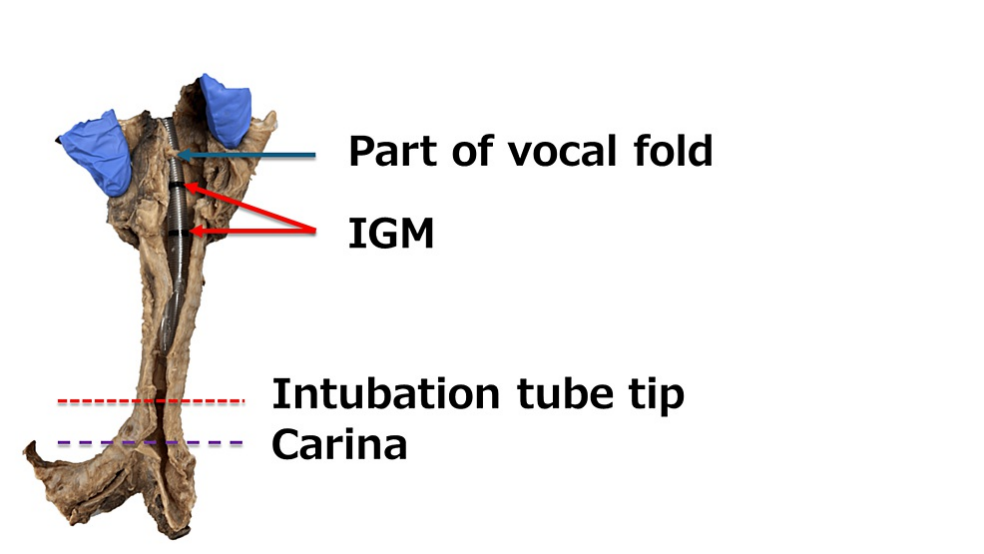
Trachea and intubation tube 2. When the tip of the intubation tube was fixed 2 cm above the tracheal bifurcation, the glottal marker (IGM) was located below the vocal folds. IGM: Intubation Guide Mark

The trachea was removed and a longitudinal incision was made in the midline on the posterior surface, maintaining the tortuous trachea’s shape. Tracheal deviation was confirmed when the chest cavity was opened, so a soft spiral tube (ShileyTM Oral/Nasal Tracheal Tube Cuffed 6.5mm I.D., COVIDIEN) was selected. When a tube was inserted and fixed at the proximal end of the glottis marker (IGM: Intubation Guide Mark), the tube tip contacted the trachea wall (Figure 4). The IGM’s position was examined when the tube tip was fixed 2 cm above the tracheal bifurcation. The trachea was stretched straight for accurate caliper and ruler measurements. The tip of the tube was fixed 2 cm above the tracheal bifurcation, and the IGM was located below the vocal cord folds (Figure 5).

## Discussion

In the present case, the trachea was compressed and deformed by the BCA and RCCA meandering. In a previous case report, we experienced cases of right common carotid tortuosity and thickening of the BCA that could have complicated a stellate ganglion block, but the cadavers did not have brachiocephalic tortuosity or tracheal deviation [7]. However, this case revealed that the trachea may be compressed and deviated when aortic meandering is aggravated. Although aortic meandering is difficult to quantify, it can be confirmed as an image using three-dimensional ultrasound [7].

Carotid tortuosity, observed here, is a common clinical condition in which the brachiocephalic or carotid artery meanders and elongates, appearing as a pulsatile neck mass [7]. These arteries are an anatomic variant of unknown cause and are typically discovered incidentally during surgery or neck imaging. They are rarely observed as a neck mass [8]. Carotid tortuosity is more common in women than in men, is anatomically predominant on the right side, and is often associated with aging, obesity, hypertension, atherosclerosis, and cardiac hypertrophy [9]. A palpable pulse in the right supraclavicular fossa may indicate right carotid meandering, especially in women with hypertension. In these cases, a thorough examination and appropriate intraoperative and postoperative approaches should be considered [7].

As shown in Figure 4, tracheal intubation at the proximal end of the IGM allows the tube tip to come into contact with the tracheal wall, suggesting possible ventilatory difficulties during intubation. To avoid these complications, we examine the appropriate tube position and the allowable range of motion based on the preoperative chest radiographs and planned surgical procedure and then use a video laryngoscope to ensure glottic visibility during intubation. After tracheal intubation, it is considered necessary to confirm the position of the tube tip using a fiberscope and place it in an appropriate position. Repeatedly verifying the tube tip position with chest X-rays should be avoided to prevent radiation overexposure.

Several factors contribute to tracheal injury associated with intubation, including older age, being female [10], forced intubation technique, inappropriate tube size, improper stylet use, an overinflated cuff, and abnormal tube position [11]. A detailed airway management plan must be developed and a spiral tube softer than polyvinyl chloride should be used to avoid tracheal injury in older women with deviated tracheas. Furthermore, as shown in Figure 5, the IGM was positioned below the vocal folds when the tube tip was fixed 2 cm above the tracheal bifurcation, which is the ideal position for the tube tip. In a patient with significant tracheal deviation, the usual tracheal intubation methods that confirm that the IGM enters the glottis cannot be used to properly position the tube tip.

Surgical airway management, a last resort during difficult intubation, requires careful planning. In the present case, as the thyroid cartilage, cricothyroid cartilage, and thyroid gland could be dissected and there were no abnormalities around the cricothyroid mesentery, cricothyroid puncture, and incision were considered anatomically and clinically feasible. However, this case was an obese woman, and when the skin was removed, there was a lot of subcutaneous fat, making it difficult to palpate the cricothyroid membrane [12].

In this case, the BCA passed in front of the trachea, suggesting a risk of aortic bleeding during percutaneous tracheostomy and tracheo-innominate artery fistula (TIF) post-surgery. Surgical procedures in the anterior neck (e.g., percutaneous tracheostomy) in patients with high common carotid or brachiocephalic arteries carry a high risk of aortic injury. A three-dimensional analysis is needed in these situations, if possible [9]. TIF is a serious complication post-tracheostomy accompanied by massive tracheal bleeding that is reported in 0.6% of patients three to four weeks after surgery [13]. Approximately 30-50% of cases experience hemorrhage before the outbreak [13]. The prognosis is poor, with a survival rate of 10-30% [13]. Anatomical risk factors include inferior tracheostomy, history of surgery or congenital scoliosis, and deviation of the trachea and BCA due to chest deformity [13]. If an anatomic abnormality is suspected, as in the present case, preoperative imaging by computed tomography should be performed to confirm the anatomic relationship between the trachea and BCA before the tracheostomy procedure [13,14].

## Conclusions

Here, we present a case of tracheal deviation due to meandering of cervical aortas to highlight potential difficulties in airway management. Tracheal deviation should be considered as a differential diagnosis when unexpected ventilation difficulties are encountered during tracheal intubation. In performing airway management, it is important not only to confirm the presence or absence of tracheal deviation using preoperative chest radiographs but also to confirm the anatomical relationship between the trachea and carotid artery.
